# Dr. Wilmer's Case of Periodic Catalepsy

**Published:** 1812-09

**Authors:** Bradford Wilmer

**Affiliations:** Coventry


### Medical and Physical Journal.

3 OF VOL. XXVIII.] SEPTEMBER, 1812. [NO. 163.

*To the Editors of the Medical and Physical Journal.*

GENTLEMEN,

THE almost exploded theory of the ancient physicians respecting the influence of the solar system in the production or phenomena of diseases, having, by the recent publications of Dr. Balfour, again engaged the attention of medical men, I am induced to send you the history of a case of periodic catalepsy, which, if sol-lunar influence is not allowed, must be regarded as a very singular coincidence of events. The description of it is a literal transcript of notes taken at the time of my attendance. It seems to support Dr. Balfour's opinions more than any of the cases published by Mead, which are chiefly taken from the writings of others. I am, Gentlemen,

*Coventry,* Your very obedient Servant,

*June* 25, 1812. BRADFORD WILMER, M.D.

March 5, 1806,—I was desired to visit Miss Hume, of Bilton Grange, in the county of Warwick, and received from Mr. Bucknell, of Rugby, the family surgeon, the following account.—Miss H. is now five months old. She was a remarkably fine child; and, at the first month, without any apparent cause, was seized suddenly with a universal spasm, which fixed all the muscles of the trunk and extremities, and for nearly forty minutes she remained as immoveable as a statue. On that day month, precisely at the same hour in the morning, she was affected in a similar manner, and the duration of the paroxysm was longer than before. In the third and fourth month the spasms returned, and with so much regularity, *recurring on the day the moon was in the full*, and so exactly as to the hour, that her parents anticipated the attack with much anxious and painful expectation. On the day before I saw her, the fit commenced about the usual hour in the morning, and went off nearly at the same period as before. After an interval of an hour it returned, and, in the course of twenty-four hours, ten of these attacks had been sustained.

The fits were usually preceded by flatulence, and some distention of the stomach. They commenced soon after waking from sleep. The abdominal muscles first took on the spasmodic action; then those of the chest, neck, face,

 head, head, and, lastly, those of the extremities. Sometimes the *flexor* muscles were first afflicted, but this was not always the case, for the fingers were sometimes forcibly contracted, or strongly extended; the head sometimes drawn towards the breast, and in other paroxysms incurvated towards the spine; but, in whatever way they were first fixed, the antagonist muscles could not (nor was any force that was used equal to) alter the position. All this time, the action of the heart and lungs was diminished; the pulse became quick and extremely feeble; respiration much interrupted, and at intervals so much suspended, that the attendants thought her dying. Her face was of a livid complexion, and a viscid mucus collected at the corners of her mouth. At length, after an uncertain time, generally from forty to sixty minutes, her breathing was less confined, the livid complexion gradually diminished, the spasmodic action of the muscles ceased, and from apparent fatigue she soon sunk into a sound sleep.

No tumor of the gums indicated the approach of teeth; she had been weaned about six weeks, since which her diet had been chiefly milk and vegetables, and her stools had always been regular, and of the usual color.

Applicetur Scrobiculo Cordis Emplastrum e Galbano.

℞. Calomel gr. ij. Mosch. gr. ii. Mf. Pulvis, quamprimum e vehiculo idoneo, sumendus.

℞. Sodæ pptt. Magnesiæ ustæ āā ℈ij. Syr. Rosæ ℥ſs. Aquæ Rosæ ℥ij. fiat Mistura cujus.

Capiat unciam dimidiam horis singulis quartis.

Habeat semicupium ex Decocto Rorismarinæ paratum.

March 8th. There has been no recurrence of muscular spasm since the last report; but her stomach has at intervals been swelled and disordered, and her breathing frequently much interrupted. After taking the powder, she passed many stools, some of which were clay-colored.

℞. Calomel gr. ij. Rhei pulv. gr. iij. Pulv. Zinzib. gr. ij. Mf. Pulvis, alternis auroris sumendus.

Continuetur misturæ.

12th. While Mr. Bucknell was present, she was seized with a fit at the usual hour. It began with difficulty of breathing: the act of inspiration was suspended nearly two minutes. Spasms began in the throat, descending to the thorax, and thence propagated through the whole system. The flexor muscles of the arms and hands were so strongly contracted, feeling as hard as a board, that they could not be extended; the face became almost black; the globes of the eye were drawn obliquely upward and outward. This attack lasted only five minutes. Mr. Bucknell applied a blister blister to the back, and ordered a mixture with valerian and asafœtida.

March 13th. The fit returned again at the accustomed hour, continuing only five or six minutes; thus, though they appear to be increased in strength, they are of shorter duration.

℞. Calomel gr. ii. Mosch. gr. ij. Cons. q. s. f. Pilula alternis diebus sumenda.

℞. Ol Ricini ʒij. (vitel ovi subact) adde.

Misturæ Moschi ℥ij. Sp. Ammoniæ fetid. ʒſs.

M. Sumat coch. parvulum horis singulis quartis.

℞. Asafœtid. ℈ij. Tinct. Opii gutt. v. Mucilag. G. Arab. ℥ſs. Aquæ puræ ℥ij. Mf. Enema, horâ septima omne mane injiciendum.

14th. She has passed a quiet night; after the injection she slept well, and has had no return of the fit.

16th. Mr. Bucknell transmitted the following letter:

"Our little patient has had no return of fits since I saw you, nor since she took the Mosch. Medicine in the last form. She has had frequent attacks of flatulence, but they have gone off without producing fits. The fetid glysters appear to be of great service: they always bring off a quantity of wind, and a copious stool, which consists of much coagulated milk. The first brought away two stools colored with bile. I have not repeated the calomel, as Mr. and Mrs. H. are afraid of its effects. At times she has been affected with spasms in her left side and leg. She appears to have less flatulence every day. If you wish me to make any alteration in the treatment, or to repeat the calomel, you will please to let me know it. "Your's, &c.

"S. Bucknell."

To this I replied,—

"I do not wish to press the further employment of calomel, nor can it be much longer continued without danger of affecting the mouth. I am of opinion also, that now we can only expect advantage from it as an evacuant, a service which may be obtained with more safety from other quarters. The great indication is to keep the bowels very open, and, as the glysters have the double effect of procuring evacuation, and obviating spasmodic action, I request that they may be continued. I have suspected coagulated milk, and the immediate cause of the disease appears to have arisen from an accumulation of acid acrimony in the stomach and intestines, acting upon a nervous system under the influence of morbid idiosyncrasy. I wish to continue the Mosch, in the following form:

 ℞. Saponis

℞. Saponis Venet. ras. ℈ij. Mellis ʒij.

Vitelli ovi q. s. Natron ppt. ʒſs.

Misturæ Moschi ℥ij. fiat Mistura cujus sumat unciam dimidiam ter quotidie.

20th. During the last four days, spasms have frequently returned, affecting chiefly the muscles of the larynx and os hyoides, and sometimes so violent as to produce an appearance of external constriction, a livid countenance, and a sonorous inspiration resembling croup. She is still costive.

Applicetur Nuchæ Emplastrum Cantharides.

℞. Kali pptt. gr. xv. Syr. Rosæ ℥ſs. Tinct. Jallapii ʒiſs.

Mist. Moschi ℥ij. fiat Mistura sumat unciam demidiam ter quotidie.

On account of an excoriation of the parts, it was necessary for a time to discontinue the glysters.

26th. Better in all respects, and the return of spasms less frequent and less violent.

30th. In two days more the period will arrive when the grand attack may be expected. In order, therefore, to prepare for it, and to lessen the morbid irritability of the part where the disease appeared to originate, and of the system in general, I directed as follows:

℞. Asafœtid. ℈ij. Tinct. Opii, gutt. x. Mucilag.

G. Arab. ℥ſs. Aquæ Puræ ℥ij. Mf. Enema horâ Septima matutina injeciendum.

Repetatur ad quatuor vices.

℞. Opii Pur. Camphoræ aa ʒj. Theriac. Androm. ℥ſs.

Mf. Pasta. Applicetur Scrobiculo Cordis.

April 2d. This being the day preceding that on which the fit was expected, Mr. Bucknell was desired to sleep in the house. The next day passed over, and no particular occurrence took place.

9th. Some return of spasm was perceived, but so slight that, had not the attendants been accustomed to see them, they would not have been noticed. From this time she recovered a firm state of health. She afterwards cut her teeth without much inconvenience. Soon after, she received the vaccine inoculation, succeeded by the hooping cough. More than six years have now elapsed without any return of the cataleptic affection, and Miss H. is at this time in perfect health.

